# YB1 and its role in osteosarcoma: a review

**DOI:** 10.3389/fonc.2024.1452661

**Published:** 2024-10-21

**Authors:** Feipeng Wu, Dapeng Li

**Affiliations:** Affiliated Hospital of Jiangsu University, Zhenjiang, Jiangsu, China

**Keywords:** osteosarcoma, splicing factor, YB1, treatment of osteosarcoma, YBX1 phosphorylation

## Abstract

YB1 (Y box binding protein 1), a multifunctional protein capable of binding to DNA/RNA, is present in most cells and acts as a splicing factor. It is involved in numerous cellular processes such as transcription, translation, and DNA repair, significantly affecting cell proliferation, differentiation, and apoptosis. Abnormal expression of this protein is closely linked to the formation of various malignancies (osteosarcoma, nasopharyngeal carcinoma, breast cancer, etc.). This review examines the multifaceted functions of YB1 and its critical role in osteosarcoma progression, providing new perspectives for potential therapeutic strategies.

## 1 Introduction

Osteosarcoma originates from mesenchymal stem cells (MSCs) and is the most common primary malignant bone tumor, predominantly occurring in children and adolescents (1). It commonly affects areas of rapid growth such as the distal femur and the proximal humerus, possibly related to hormonal changes during puberty (2). Most cases of osteosarcoma invade surrounding tissues and metastasize, commonly to the lungs. With the introduction of chemotherapy, the 5-year survival rate has increased to about 70%, but the rate drops to 20%-30% for recurrent or lung-metastasized cases, highlighting the urgent need for novel therapeutic targets to enhance patient survival rates (3, 4).

RNA splicing, the process of removing non-coding introns from precursor RNA to produce mature mRNA, is a dynamic and complex process (5). Approximately 95% of gene splicing involves alternative splicing, where certain introns are selectively removed during the RNA splicing process. This mechanism allows a single gene to produce multiple mature mRNAs, thereby enabling the efficient realization of multiple functions by human genes (6, 7).

Alternative splicing plays a crucial role in the development of osteosarcoma, particularly through mechanisms that regulate the immune microenvironment. Research has identified several alternative splicing events associated with osteosarcoma, which may promote tumor progression by affecting the function of key immune cells, such as CD8+ T cells and dendritic cells. Additionally, RNA-binding proteins (RBPs) play a significant role in regulating these splicing events, suggesting that they may serve as potential therapeutic targets (8).

The splicing process is highly regulated, mediated by the spliceosome, a macromolecular complex composed of ribonucleoproteins (RNPs) and small nuclear RNA (snRNA), which recognizes intron 5’ GU and 3’ AG splice sites (9). However, in addition to regulation by splice sites and the spliceosome, the splicing process is also regulated by cis-regulatory elements and trans-acting factors, Cis-regulatory elements are specific RNA sequences located on precursor RNA, including exon splicing enhancers (ESE), intron splicing enhancers (ISE), and exon splicing silencers (ESS). Their primary function is to recruit trans-acting factors (splicing factors) to specific sites, thereby precisely regulating the activation or inhibition of splice sites by the spliceosome (10–12). With the deepening of research on the RNA splicing process, increasing evidence suggests that abnormal RNA splicing plays a crucial role in various diseases, especially cancer (13, 14). Here are some examples of how the splicing process affects osteosarcoma: Serine/Arginine-rich Splicing Factor 1 (SRSF1) is an important splicing factor that has been found to be closely associated with the progression of osteosarcoma when highly expressed. SRSF1 regulates the splicing of multiple genes, including HIF-1α and VEGF-A, which play crucial roles in angiogenesis, cell proliferation, and anti-apoptotic pathways. Additionally, SRSF1 may influence the function of tumor-infiltrating T cells by regulating the splicing of genes related to immune suppression, thereby helping tumor cells evade immune surveillance. Through these splicing events, SRSF1 enhances tumor cell invasiveness and drug resistance, further driving the progression of osteosarcoma (8, 15). Small extracellular vesicles (sEVs) derived from osteosarcoma cells influence the splicing factors by carrying and delivering specific miRNAs and proteins. These sEVs modulate the splicing of genes related to Notch2 and GATA3, further promoting tumor cell metastasis (16).

**Figure 1:**
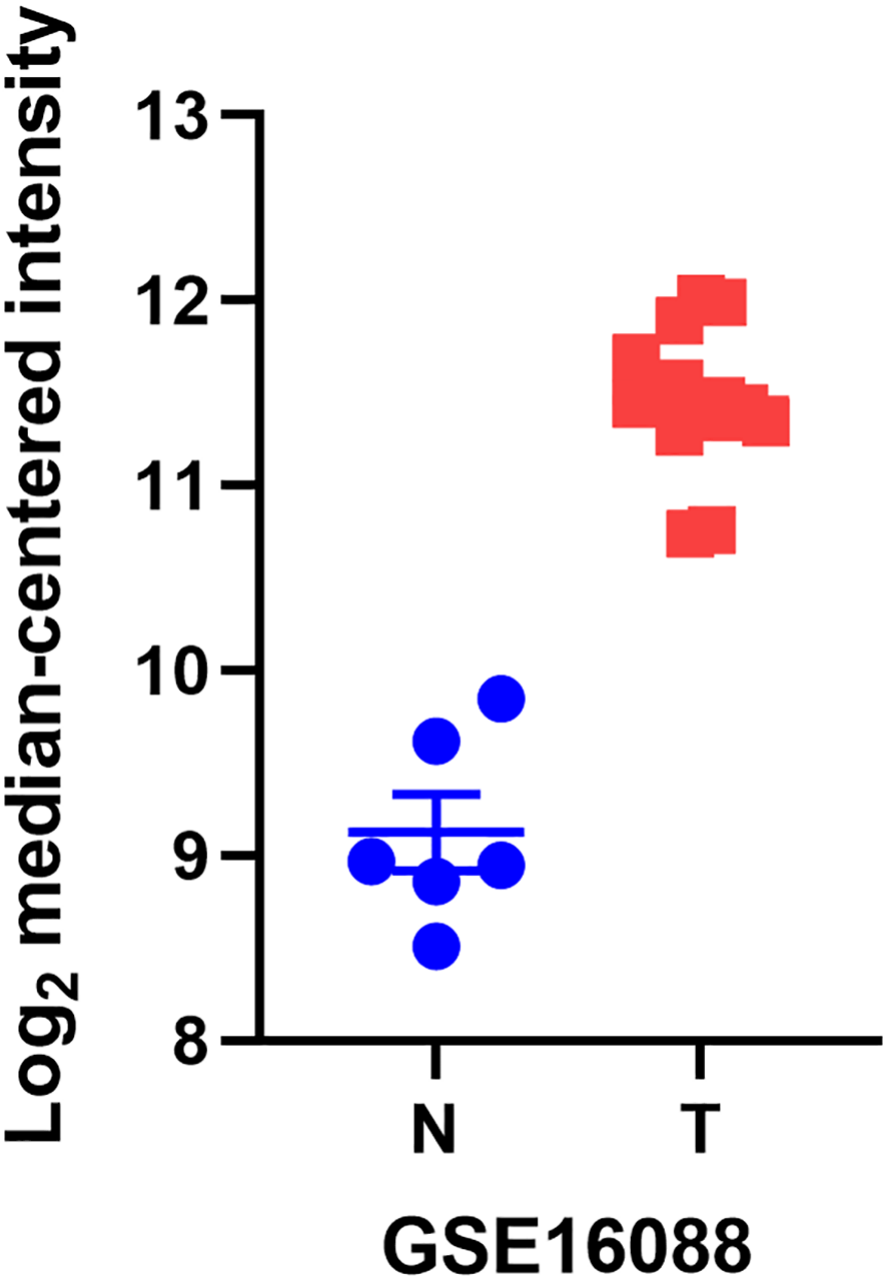
The differential mRNA expression of YB1 in normal kidney, liver, and lymph node tissues compared to osteosarcoma.

YB1, also known as DNA binding protein B (DbpB), often acts as a splicing factor in cellular regulation and plays a key role in a wide range of cellular functions. Its abnormal expression has a significant impact on tumor formation (17, 18). We found through the GEO database that YB1 is more abundant in osteosarcoma tissues compared to normal tissues (**Figure 1**). This article systematically introduces the molecular structure and functions of YB1 and its relationship with osteosarcoma, providing new targets for the treatment of this cancer.

## 2 Structure and expression of YB1

### 2.1 Molecular structure of YB1

**Figure 2:**
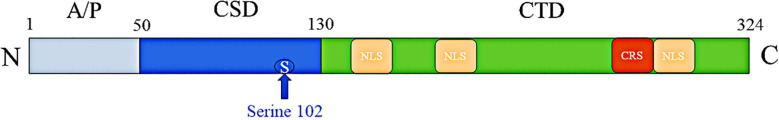
The structural domain diagram of YB-1.

YB-1 is a 42 kDa protein, primarily known as a specific DNA-binding protein and a member of the cold shock domain (CSD) protein superfamily. It was initially discovered for its ability to recognize and bind the Y box sequence 5′-CTGATTGGCCAA-3′ (an inverted CCAAT box) in the promoter of MHC class II genes. Subsequently, YB1 was found to be a major component of messenger ribonucleoprotein particles in the cytoplasm, interacting with mRNA (19, 20). The YBX1 gene, which encodes YB1, is located at 1p34 on chromosome 1 (21). It has three functional domains (**Figure 2**): a variable N-terminal domain rich in alanine and proline (A/P domain); a centrally located evolutionarily conserved CSD protein, which can bind nucleic acids specifically or non-specifically; and a hydrophilic C-terminal region (CTD), which can bind nucleic acids and many proteins (22, 23). The CTD regulates the distribution of YB1, containing atypical nuclear localization signals (NLS) and cytoplasmic retention signals (CRS). Under normal circumstances, CRS dominates over NLS, thus YB1 is mainly located in the cytoplasm (24, 25).

### 2.2 Expression of YB1

YB1 expression is dynamically regulated during developmental stages and in various tissue states. Its crucial role in embryonic development is underscored by the lethality observed in ybx1 gene knockout mouse embryos (26–28). As age increases, its expression declines, and its abnormal expression can serve as both a prognostic factor and a target for cancer therapy (24, 29, 30). For example, YBX1 is not expressed or maintained at very low levels in normal adult liver, but its expression increases again during fetal development or liver injury repair, as well as in liver cancer (31). Studies have shown that overexpression of YBX1 is associated with various tumor types’ resistance to therapy, cell proliferation, and prognosis, such as breast cancer, esophageal cancer, lung cancer, sarcomas, prostate cancer, glioblastoma, and non-Hodgkin lymphoma (32, 33). The expression of YB-1 is also regulated by other factors. At the transcriptional regulation level, certain transcription factors, DNA methylation, and histone modifications, as well as hypoxic environments, can affect its transcription. At the translational level, factors such as the structure of the mRNA and specific miRNAs can impact YB-1 translation. At the post-transcriptional level, the stability of YB-1 is influenced by various post-translational modifications, such as phosphorylation and ubiquitination. These modifications may affect the degradation and function of YB-1, thereby influencing its biological roles within the cell (34–37).

## 3 The role of YB1 in normal physiological functions

### 3.1 Regulation of bone marrow stromal cell (BMSC) activity

**Figure 3:**
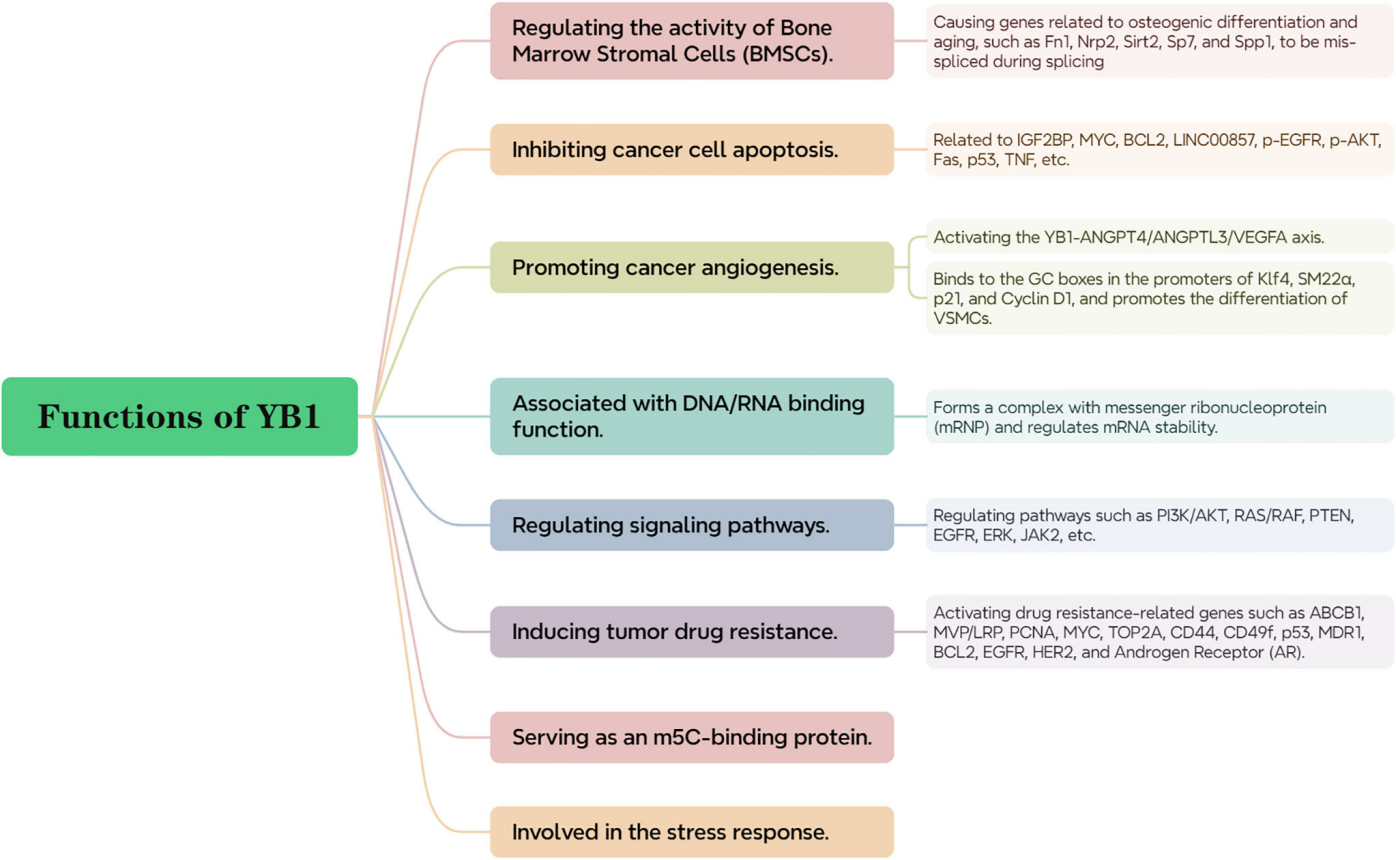
The functions of YB1.

Alternative splicing (AS) as an important post-transcriptional regulatory pathway contributes to gene expression diversity and is related to the aging of BMSCs in humans and mice. The expression of splicing factor YB1 in BMSCs declines with cell aging. The loss of YB1 can lead to erroneous splicing of genes related to osteogenic differentiation and aging (such as Fn1, Nrp2, Sirt2, Sp7, and Spp1), thereby promoting the aging and bone loss of BMSCs, while stimulating overexpression of YB1 inhibits BMSC aging and promotes bone formation (38) (**Figure 3**).

### 3.2 DNA/RNA binding function

YB1 was initially identified as a transcription factor due to its ability to bind and enhance the activity of major histocompatibility complex (MHC) class II and epidermal growth factor receptor (EGFR) genes. In the cell nucleus, it acts as a transcription factor, while in the cytoplasm, it forms complexes with messenger ribonucleoproteins (mRNPs) and regulates mRNA stability (34). Studies have shown that YB1 acts as a transcriptional regulator involved in the transcriptional regulation of genes related to cell proliferation such as E2F1. Phosphorylated YB1 is an important regulator for RNPs, as unphosphorylated YB1 binds to translationally inactive mRNPs, inhibiting internal ribosome-dependent mRNA translation. However, once YB1 is phosphorylated at serine 102 (S102), its ability to bind mRNA is reduced, allowing mRNA translation and thereby regulating cell proliferation (39). For example, ribosomal S6 kinase 2 (RSK2) mediated phosphorylation of YB-1 at the Ser102 site promotes the formation of the YB-1/KLF5 transcription complex, which jointly regulates the expression of BLBC-specific genes such as keratin 16 (KRT16) and lymphocyte antigen 6 family member D (Ly6D), promoting cancer cell proliferation (40) (**Figure 3**).

### 3.3 Signal pathway regulation

YB1 promotes osteogenic differentiation of bone marrow MSCs by activating the PI3K/AKT pathway and can also lead to activation of the PI3K/AKT pathway by reducing the expression of the tumor suppressor gene PTEN (41, 42). Moreover, YB1 can control the mutation state of RAS/RAF, thereby regulating cell apoptosis and EGFR expression (43). Evidence suggests that the PI3K/AKT and RAS/RAF pathways are related to cancer formation, so YB1 is inextricably linked with cancer; YB1, through its role as a splicing factor, participates in regulating RNA splicing processes, thereby affecting the ERK signaling pathway; YB1 has been found to be a key factor in the persistence of JAK2 mutant tumors. Studies have shown that inactivation of YB1 increases cell sensitivity to JAK2 inhibitors, thereby promoting cell apoptosis. This finding reveals the important role of YBX1 in the survival and proliferation of JAK2 mutant cells, pointing out a potential therapeutic target, i.e., targeting YBX1 to enhance the efficacy of JAK2 inhibitors, providing a new strategy for treating JAK2 mutant-related diseases (6) (**Figure 3**).

### 3.4 Serving as an m5C binding protein

Methylation is the most common form of RNA modification, and 5-methylcytosine (m5C) is a post-transcriptional RNA modification involved in the progression of many cancers (44). However, YB-1 has been defined as an RNA m5C binding protein (45). Recent studies have shown that YB-1 preferentially binds m5C RNA through interactions with two tryptophan residues (Trp45 and Trp65) in the CSD, which is particularly important in the progression of diseases such as bladder cancer, as YB-1 overexpression is associated with tumor proliferation and metastasis. Specifically, YB-1 promotes cancer cell proliferation by stabilizing specific m5C-modified mRNAs, such as HDGF and KLF5 mRNA. Additionally, YB-1 also plays a role in stabilizing m5C-modified maternal mRNAs during early embryonic development in zebrafish and in the development of adult stem cells and the maintenance of germline stem cells in fruit flies (46, 47) (**Figure 3**).

### 3.5 Involvement in cellular stress responses

Of particular interest is YB-1’s role in the cellular stress response, especially in the formation of stress granules. These are non-membranous organelles that form in response to environmental stressors such as heat shock, oxidative stress, or viral infections. Stress granules consist predominantly of untranslated mRNAs and associated proteins, serving as protective storage sites for these molecules. YB-1 is implicated in both the assembly and regulatory functions of stress granules, which are vital for cell survival under stress conditions (48) (**Figure 3**).

These aspects are critical in understanding the multifaceted roles of YB-1 in cell biology. If these elements are not addressed in the review, it could lead to an incomplete understanding of YB-1’s functions and its significance in various cellular contexts.

## 4 The role of YB1 in cancers

### 4.1 Inhibition of cancer cell apoptosis

Studies have shown that low expression of YB1 promotes apoptosis of laryngeal squamous cell carcinoma (LSCC) cells and glioma stem cells, inhibiting cancer cell proliferation and metastasis (49, 50). YB1 can interact with insulin-like growth factor receptor 2 messenger RNA binding protein (IGF2BP) and stabilize N6-methyladenosine (m6A) modified RNA such as MYC and BCL2 mRNA, supporting the survival of myeloid leukemia cells, although YB1 does not appear to affect normal hematopoietic function (51). YB1 interacts with the potential oncogene LINC00857, preventing its degradation by the proteasome and increasing its nuclear translocation, and also regulates the protein expression of p-EGFR, p-AKT, and their downstream target genes, thus affecting cell apoptosis (52–54). YB-1 may also act as a transcriptional inhibitor of the cell death-related Fas receptor gene and can directly regulate the expression and activity of the apoptosis regulator p53 to inhibit cancer cell apoptosis (55). Cells deficient in YB1 seem to be more susceptible to TNF-induced apoptosis (56) (**Figure 3**).

### 4.2 Promotion of cancer angiogenesis

In mouse models, YB1 in endothelial cells can upregulate the motility of endothelial cells and the secretion of angiogenic factors in extracellular vesicles (including exosomes), thus playing a crucial role in endothelial cell-induced tumor formation and angiogenesis and YB-1 is transferred to human umbilical vein endothelial cells (HUVECs) via gastric cancer cell-derived exosomes, directly promoting cancer angiogenesis by upregulating key angiogenic factors (57). YB1 can interact with linc00665 and activate the YB1-ANGPT4/ANGPTL3/VEGF axis to induce tumor-related angiogenesis in LUAD (58);YB1 can also bind to the GC boxes in the promoters of Klf4, SM22α, p21, and cyclin D1, promoting VSMC differentiation. Since VSMCs are major components of the vascular wall and play a crucial role in vascular formation, remodeling, and angiogenesis, they further promote cancer angiogenesis (59, 60). Using antisense oligonucleotides targeting YB1 (YB1-ASOA) can specifically inhibit YB-1 protein expression in tumor vascular endothelial cells, thereby downregulating the Bcl-xL/VEGFR2 and Bcl-xL/Tie signaling axes, inhibiting these two key angiogenic regulatory factors, reducing angiogenesis, and thereby inhibiting tumor growth, which also indirectly illustrates YB1’s role in promoting cancer vascular growth (61) (**Figure 3**).

### 4.3 Induction of tumor drug resistance

YB1 acts as a transcriptional activator, promoting the expression of multiple genes associated with drug resistance. This includes genes such as MDR1, MVP/LRP, MYC, CD44, BCL2, PCNA, TOP2A, p53, CD49f, ABCB1, and androgen receptor (AR). This upregulation contributes to the ability of cancer cells to resist various anti-cancer treatments. The expression of YB1 is usually associated with resistance to platinum drugs (such as cisplatin) and also promotes the malignant progression of cancer cells and acquisition of drug resistance by regulating genes involved in cell proliferation and the cell cycle, such as EGFR and HER2 (62–66); targeting the AKT/mTOR/p70S6k or MEK/ERK/p90RSK signaling pathways to inhibit downstream YB1 activity can inhibit tumor drug resistance (67) (**Figure 3**).

## 5 The role of YB1 in osteosarcoma

### 5.1 YB1 regulation of VEGF promotes osteosarcoma progression

Vascular endothelial growth factor (VEGF) is highly expressed in osteosarcoma and can act as a paracrine molecule in the tumor microenvironment to promote tumor angiogenesis, as well as an autocrine molecule to promote tumor proliferation and metastasis, thus promoting the occurrence and development of osteosarcoma and being related to the prognosis of osteosarcoma. Among these, VEGF165 and its homologous isoform VEGF165b, although identical in length, have completely opposite effects; the former promotes cancer while the latter inhibits it. As a splicing factor, YB1 modulates the expression of VEGF165 and VEGF165b, leading to increased VEGF165 levels and decreased VEGF165b expression. This alteration in VEGF isoform balance promotes osteosarcoma cell formation and metastasis (68, 69).

### 5.2 YB1 interaction with RNA affects osteosarcoma progression

Circular RNA (circRNA) can regulate gene expression in many malignancies by capturing microRNAs (miRNAs). Studies have shown that circ0001658 and YB1 are highly expressed in osteosarcoma and promote the proliferation and metastasis of osteosarcoma, while miR-382 is expressed very little, inhibiting the progression of osteosarcoma. The promotion of osteosarcoma development by circ0001658 is related to its regulation of miR-382-5p/YB1 expression. Circ0001658 negatively regulates miR-382-5p while positively regulating YB1 expression, thereby promoting the occurrence and development of osteosarcoma. In contrast, miR-382 targets and inhibits YB1 expression, exerting its inhibitory effect on osteosarcoma progression (70, 71).

### 5.3 The critical role of YB-1 in activating HIF-1α during osteosarcoma progression

Hypoxia or low oxygen is a common feature of solid tumors, including sarcomas (72). To cope with hypoxic conditions, tumors initiate transcription and other processes to adapt to this environment. Hypoxia promotes cancer progression and metastasis, among which the key molecule is tumor hypoxia-inducible factor 1 (HIF-1). Under hypoxic conditions, the expression of HIF-1 increases, and it participates in carcinogenesis and tumor growth by regulating genes involved in angiogenesis, glycolytic metabolism, and other biological mechanisms (73). Cancer cells switch their metabolism from oxidative phosphorylation to aerobic glycolysis under hypoxic conditions, producing large amounts of lactate, a process known as the Warburg effect, which is directly related to HIF-1. Enhanced glycolysis not only meets the ATP demand for rapid tumor growth but also provides sufficient metabolic intermediates for rapid tumor proliferation (74, 75). El-Naggar and colleagues found that YB-1 is a key regulator of hypoxia-inducible factor 1α (HIF1α) expression in sarcoma cells. YB-1 binds to the IRES sequence in the 5′ UTR region of HIF1-α mRNA, activating HIF-1α at the translational level, thereby enhancing the invasion and metastatic capabilities of sarcoma cells, highlighting the importance of the translationally regulated YB-1-HIF1α axis in sarcoma metastasis (37, 76–78).

### 5.4 YB1 promotes osteosarcoma cell proliferation

YB1 can promote the transcription of cell cycle proteins (D1, A, B1), enhancing cancer cell proliferation. *In vitro* and *in vivo* experiments using specific siRNAs to inhibit YB1 expression have also inhibited the expression of cell cycle proteins D1 and A, key regulatory proteins for the G1/S phase transition of the cell cycle, thereby inhibiting the proliferation of osteosarcoma cells (79–81). Studies have shown that YB1 not only affects the G1/S phase transition of the cell cycle but also affects the G2/M phase transition. Silencing YB1 leads to G2/M phase cell cycle arrest (82).

## 6 The role of YB1 in other tumors

In non-small cell lung cancer, the lncRNA HOXC-AS3 directly interacts with YB1 and inhibits YB1 ubiquitination, thereby reducing YB1 degradation and increasing YB1 levels. HOXC-AS3 and YB1 also promote lung cancer proliferation and metastasis by increasing the amount of HOXC8 (83). In nasopharyngeal cancer, Circlpo7 interacts with cytoplasmic YB1, promoting the phosphorylation of YB1 at S102, further promoting YB1’s nuclear translocation, and activating FGFR1, TNC, and NTRK1 transcription, thereby promoting nasopharyngeal cancer metastasis and resistance to cisplatin (84). YB1 can bind to the promoter region of cytidine nucleotide triphosphatase (CTPS1) in triple-negative breast cancer (estrogen receptor-deficient, progesterone receptor-deficient, human epidermal growth factor 2-deficient) cells and activate CTPS1 transcription, increasing CTPS1 expression, and thereby promoting breast cancer proliferation and metastasis (85). The long non-coding RNA (lncRNA) AWPPH is highly expressed in liver cancer and indicates a poor prognosis. At the same time, the interaction between lncRNA AWPPH and YB1 promotes liver cancer cell proliferation and metastasis (86). The lncRNA lnc-SOX9-4 can increase YB1 levels, thereby enhancing the proliferation and metastasis capabilities of colorectal cancer (CRC) cells (87). YB1 is also significantly related to many other tumors, which are not all listed here.

## 7 Research on YB1 as a target for treating osteosarcoma

How to target and inhibit YB1 to treat osteosarcoma is a current challenge. Studies have shown that miR-382 can target and inhibit YB-1 expression at the 3′UTR, thereby inhibiting the proliferation, invasion, and epithelial-mesenchymal transition of osteosarcoma cells. Simultaneously, the combination of miR-382 with chemotherapeutic drugs such as doxorubicin can inhibit osteosarcoma growth and recurrence and reduce the proportion of cancer stem cells (CSC), making miR-382 a potential target for osteosarcoma treatment (71). Class I HDACs, such as MS-275, can enhance the ROS levels in osteosarcoma, while inhibiting YB1-mediated antioxidant regulator NRF2 expression, thereby indirectly increasing ROS levels in tumor cells. This drug can also enhance the acetylation of lysine 81 in the YB1 RNA-binding domain, thereby losing YB1-mediated translation activation, ultimately blocking the translation and synthesis of NRF2, G3BP1, and HIF1α in sarcoma cells mediated by MS-275, thereby inhibiting the progression of osteosarcoma (88). Somasekharan et al. demonstrated that YB-1 interacts with G3BP1 mRNA transcripts in an osteosarcoma model, which plays a significant role in the biology of osteosarcoma, particularly in tumor progression and metastasis. This interaction highlights how YB-1 may modulate the expression of G3BP1, influencing the invasive capabilities and overall aggressiveness of osteosarcoma cells (89). Niraparib (P1), as a PARP-1 inhibitor, has been approved for use in advanced ovarian cancer treatment. Its main mechanism is to interact with key residues in the Quercetin pocket of YB-1, such as F85 and K118, especially through π-π stacking and van der Waals (vdW) interactions, to specifically disrupt the interaction of YB-1 with mRNA, showing high selectivity for YB-1 (90).

## 8 Research on YB1 as a target for treating tumors

**Figure 4:**
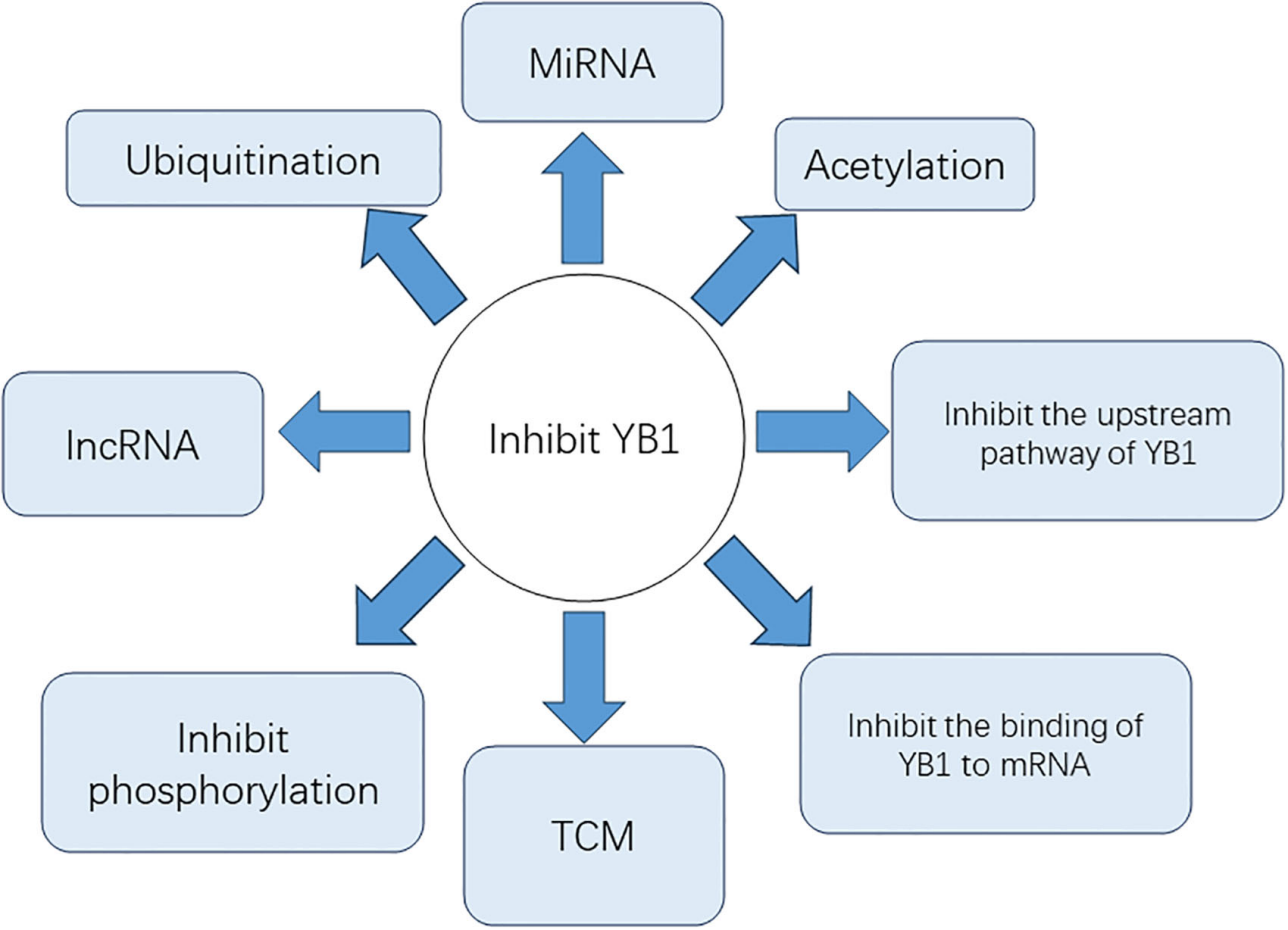
The methods for inhibiting YB1.

Strategies to inhibit YB1 and its downstream pathways are currently at the forefront of osteosarcoma research. YB1 is a protein characterized by its intrinsically disordered nature, which means it lacks a stable, fixed three-dimensional structure. This intrinsic disorder poses challenges for traditional structural biology approaches, which typically rely on stable protein conformations. The flexibility conferred by this disorder allows YB-1 to engage in diverse interactions with various RNA and protein partners, playing crucial roles in multiple cellular processes. Here are some examples for reference (89) (**Figure 4**).

### 8.1 Inhibition of YB1-related pathways

Studies have shown that the PI3K/mTOR inhibitor BEZ235 can inhibit YB1 expression and enhance the toxicity of radiation to cells. The combined treatment of BEZ235 and radiotherapy has shown strong anti-tumor effects in mouse models (91).

### 8.2 Gene interference technology inhibits YB1

The circRNA circNEIL3 (hsa_circ_0001460) and Nfix circRNA (circNfix) have shown strong anti-cancer effects in *in vitro* and *in vivo* models. CircNEIL3 directly acts on YB1, promoting Nedd4L-mediated proteasome elevation to enzymatically degrade oncogenic protein YB1, thereby reducing YB1 levels and exerting an inhibitory effect on cancer metastasis (92, 93). The lncRNA LINC00472 interacts with YB1, inhibiting the epithelial-to-mesenchymal transition of epithelial cells and affecting the mechanical properties of cancer cells, thereby inhibiting the invasion and metastasis capabilities of lung cancer cells (94). MiR-216a can directly bind to the 3′ untranslated region of the YB-1 gene to inhibit YB-1 expression, thereby inhibiting the invasion and metastasis of pancreatic cancer (95). Linc01612 promotes the ubiquitination and proteasome pathway degradation of YBX1, thereby reducing YB1 protein stability, reducing YB1 protein levels, but not affecting its RNA expression levels, exerting its inhibitory effect in hepatocellular carcinoma (36). SiRNA knockdown of Ybx1 can reduce Ybx1 mRNA levels by about 80%. Disrupting YB1’s ability to bind to related mRNA can also inhibit tumor development (96).

It is also possible to design DNA/RNA sequences that specifically bind to YB1 mRNA and attach corresponding specific carriers to enhance the enzymatic cleavage of YB1 mRNA, thereby inhibiting YB1 (97).

### 8.3 Inhibition of YB1 phosphorylation at relevant sites

RBM10 forms a ternary complex with YB1 and PPM1B to inhibit YB1 phosphorylation, or natural products such as lacquer yellow, TAS0612/everolimus inhibit AKT/mTORC1-mediated phosphorylation of YB1 at ser102, thereby inhibiting tumor drug resistance and further development (63, 98–100). However, not all phosphorylation sites of YB1 promote tumor formation. For example, when YB-1 protein is phosphorylated by Akt kinase at serine 209 (S209), its nuclear translocation activity is inhibited. This phosphorylation prevents the nuclear import of YB-1 protein, which is otherwise facilitated by phosphorylation at serine 102 (S102) (35).

### 8.4 Direct physical interaction of SU056 inhibits YB-1 function and protein levels

SU056, a small molecule drug, directly physically interacts with YB-1, significantly reducing YB-1 protein levels and affecting various proteins and pathways related to YB-1 functions, including apoptosis, RNA degradation, and cell cycle regulation, effectively inhibiting YB-1 (101, 102).

### 8.5 Inhibition of YB1 binding to mRNA

TET2 is a DNA dioxygenase, which acts on m5C in mRNA through its DNA dioxygenase activity, oxidizing it to form 5-hydroxymethylcytosine (5hmC). TET2’s oxidation weakens YBX1’s binding affinity to mRNA because YBX1’s recognition ability for 5hmC-modified mRNA is weaker than that for 5mC, thus indirectly inhibiting YBX1’s function by weakening its binding to target mRNA, thereby reducing mRNA stability and inhibiting tumor development. Similarly, α-ketoglutarate-dependent dioxygenase ABH1 (ALKBH1) has a similar effect (103, 104).

### 8.6 Traditional Chinese medicine (TCM)

Yifei Sanjie (YFSJ), a well-applied traditional Chinese medicine formula, can inhibit YB1 expression, thereby inhibiting lung tumor progression (105).

In summary, significant progress has been made in the research on targeting YB-1 for the treatment of osteosarcoma. Gene interference techniques, natural products, and small molecule drugs each demonstrate distinct advantages and potential (**Table 1**). In the future, the combination of multiple strategies and combination therapies may provide more effective approaches for the clinical treatment of osteosarcoma.

**Table 1:** List of specific inhibitors of YB1.

Drugs	Functions
MiR-382	Targeted inhibition of YB-1 expression at the 3′UTR site.
Class I HDAC inhibitors such as MS-275	Enhanced acetylation of lysine 81 within the RNA-binding domain of YB-1 leads to the loss of YB-1 translational activation.
Niraparib(P1)	Disrupt the interaction between YB-1 and mRNA.
PI3K/mTOR inhibitor BEZ235	Inhibition of YB1 expression enhances the cytotoxicity of radiation to cells.
CircNEIL3 (hsa_circ_0001460) in circRNA	Promote Nedd4L-mediated proteasomal degradation to enzymatically degrade the oncogenic protein YB1.
LINC00472 in lncRNA	Binding to YB1 inhibits the epithelial-to-mesenchymal transition (EMT).
MiR-216a	It can directly bind to the 3’ untranslated region (3’UTR) of the YB-1 gene to inhibit YB-1 expression.
SU056	Direct physical interaction with YB-1 can significantly reduce the levels of YB-1 protein.
Linc01612	Promote the ubiquitination and proteasomal degradation of YBX1.
RBM10, Quercetin, TAS0612, Everolimus	Inhibit AKT/mTORC1-mediated phosphorylation of YB1 at Ser102.
SIRNA	Reduce Ybx1 mRNA levels by approximately 80%.
TET2, α-ketoglutarate-dependent dioxygenase ABH1 (ALKBH1)	Oxidation of 5-methylcytosine (5mC) to 5-hydroxymethylcytosine (5hmC) on mRNA reduces the binding affinity of YBX1 to mRNA.
Traditional Chinese Medicine Yifei Sanjie (YFSJ)	Inhibit the expression of YB1.

## 9 Outlook

In summary, the splicing factor YB1 is highly expressed in osteosarcoma cells and promotes the proliferation, metastasis, angiogenesis, and inhibition of apoptosis of osteosarcoma cells through its influence on downstream pathways. Researching YB1 helps further study the mechanisms of osteosarcoma and other tumors’ occurrence and development, thereby better treating osteosarcoma. However, there are still many issues to be resolved regarding YB1: (1) The downstream pathways of YB1 are very complex, and how YB1 affects the occurrence and development of tumors in different tumors still requires further study. (2) How to target and inhibit YB1 is a current challenge, and few drugs can precisely target and inhibit YB1. (3) Whether inhibiting YB1 will produce irreversible effects on cells other than tumor cells is unknown. (4) The mechanism of tumor onset is complex, and single-target treatment cannot effectively treat osteosarcoma. It is very urgent to seek other targets in the mechanism of osteosarcoma onset. This review offers insights into potential combinatorial therapeutic approaches targeting YB1 alongside other molecular targets, paving the way for more effective osteosarcoma treatments.
